# Identification of genes regulated by Wnt/β-catenin pathway and involved in apoptosis via microarray analysis

**DOI:** 10.1186/1471-2407-6-221

**Published:** 2006-09-07

**Authors:** Moli Huang, Yihua Wang, Daochun Sun, Hongxia Zhu, Yanbing Yin, Wei Zhang, Shangbin Yang, Lanping Quan, Jinfeng Bai, Shengqi Wang, Quan Chen, Songgang Li, Ningzhi Xu

**Affiliations:** 1 Center of Bioinformatics, National Laboratory of Genetic Engineering and Protein Engineering, College of Life Sciences, Peking University, Beijing, P. R. China; 2 Laboratory of Cell and Molecular Biology, Cancer Institute & Cancer Hospital, Chinese Academy of Medical Sciences & Peking Union Medical College, Beijing, P. R. China; 3 No.9 lab, Beijing Institute of Radiation Medicine, Beijing, P. R. China; 4 The Laboratory of Apoptosis and Cancer Biology, The National Key Laboratory of Biomembrane and Membrane Biotechnology, The Institute of Zoology, Chinese Academy of Sciences, Beijing, P. R. China

## Abstract

**Background:**

Wnt/β-catenin pathway has critical roles in development and oncogenesis. Although significant progress has been made in understanding the downstream signaling cascade of this pathway, little is known regarding Wnt/β-catenin pathway modification of the cellular apoptosis.

**Methods:**

To identify potential genes regulated by Wnt/β-catenin pathway and involved in apoptosis, we used a stably integrated, inducible RNA interference (RNAi) vector to specific inhibit the expression and the transcriptional activity of β-catenin in HeLa cells. Meanwhile, we designed an oligonucleotide microarray covering 1384 apoptosis-related genes. Using oligonucleotide microarrays, a series of differential expression of genes was identified and further confirmed by RT-PCR.

**Results:**

Stably integrated inducible RNAi vector could effectively suppress β-catenin expression and the transcriptional activity of β-catenin/TCF. Meanwhile, depletion of β-catenin in this manner made the cells more sensitive to apoptosis. 130 genes involved in some important cell-apoptotic pathways, such as PTEN-PI3K-AKT pathway, NF-κB pathway and p53 pathway, showed significant alteration in their expression level after the knockdown of β-catenin.

**Conclusion:**

Coupling RNAi knockdown with microarray and RT-PCR analyses proves to be a versatile strategy for identifying genes regulated by Wnt/β-catenin pathway and for a better understanding the role of this pathway in apoptosis. Some of the identified β-catenin/TCF directed or indirected target genes may represent excellent targets to limit tumor growth.

## Background

The Wnt/β-catenin pathway has key roles in embryonic patterning and cell-fate determination [1,2]. Defects in this pathway have also been implicated in human cancers [3-5]. It is believed that accumulation of β-catenin in the cytoplasm favors its translocation to the nucleus as a cofactor for transcription factors of the T-cell factor/lymphoid enhancing factor (TCF/LEF) family and activates the transcription of Wnt/β-catenin target genes, which regulates cell proliferation and differentiation [3]. Although significant progress has been made in understanding the downstream signaling cascade of Wnt/β-catenin pathway, the precise role of this pathway in apoptosis remains unclear. Previous studies demonstrated that inhibiting the activity of Wnt/β-catenin pathway induced apoptosis; meanwhile, activation of this pathway inhibited chemotherapy-induced apoptosis [6]. Thus, Wnt/β-catenin pathway may be associated with cellular apoptosis. Despite of these findings, the molecular mechanisms by which Wnt/β-catenin pathway regulates apoptosis are unclear until now. Chen *et al*. found that Wnt-1-mediated β-catenin/TCF transcription was responsible for providing protection against apoptosis [7]. However, so far, only a small number of target genes regulated by Wnt/β-catenin have been characterized [3]. Even less is known about transcriptionally regulated genes at the genome-wide scale that contribute to the apoptosis effect.

The advent of RNA interference (RNAi)-directed knockdown has sparked a revolution in somatic cell genetics, allowing the inexpensive, rapid analysis of gene function in mammals [8-10]. RNAi effects can be achieved by transfection of short synthetic double stranded RNA molecules or gene expression vectors that direct their production in the cell. These small interfering RNA (siRNA) expression vectors have several advantages over chemically synthetic siRNA: they can be stably introduced into cells, and they are relatively less expensive and more efficient. However, as with conventional knockout strategies, stably introduced siRNA vectors cannot be used when the target is essential for cellular survival. Recently, van de Wetering *et al*. developed a doxycycline (DOX)-inducible form of the RNA polymerase III H1 promoter to drive siRNA expression, which could inducibly knockdown the expression of target genes [11]. With the help of this inducible system, we successfully downregulated the expression of β-catenin in HeLa cells by addition of DOX to the growth medium. We found that decreased expression of β-catenin in this manner reduced the transcriptional activity of β-catenin/TCF and promoted the death of cells. To further investigate the potential mechanisms underlying the role of Wnt/β-catenin pathway in apoptosis, we designed an oligonucleotide microarray covering 1384 apoptosis-related genes. We then performed microarray analysis to visualize differential gene regulation before *vs*. after induction. A series of differential expression genes due to reduced expression of β-catenin was identified and confirmed by RT-PCR. These genes have been implicated in apoptosis but never linked to Wnt/β-catenin pathway before. Since apoptosis is critical for normal embryonic development and for homeostasis in adult tissues, cancellation of this process with increased resistance to cell death is a common feature of malignant cells and represents a significant obstacle to therapy of human cancers [12,13], some of the identified β-catenin/TCF directed or indirected target genes may represent excellent targets to limit tumor growth.

## Methods

### Cell line and tissue culture

The parental T-REx™-HeLa cells (Invitrogen, abbreviated to HeLaT), stably expressing the tetracycline repressor protein were grown in DMEM (Invitrogen) supplemented with 10% fetal bovine serum (Gibco BRL), 50 units/ml penicillin, 50 units/ml streptomycin and 5 μg/ml blasticidin. These cells were maintained in a humidified 37°C incubator with 5% CO_2_, fed every 3 days with complete medium supplemented with 5 μg/ml blasticidin, and subcultured when confluence was reached.

### Plasmids, transfection and generation of stable pTER cell line

The plasmid pTER-β-catenin, which encodes a short hairpin RNA (shRNA) against human β-catenin driven by a DOX-inducible form of the RNA polymerase III H1 promoter, was a kind gift from Dr. M. van de Wetering and Prof. H. Clevers (Hubrecht Laboratory, Center for Biomedical Genetics, Utrecht, The Netherlands) [11]. TOPFLASH, FOPFLASH were purchased from Upstate and pRL-TK was purchased from Promega.

Transfections were performed in 6-well or 24-well plates. A total of 2 × 10^5 ^cells were seeded into each well of a six-well tissue culture plate (Costar).The next day when the cells were 70–80% confluent the culture medium was aspirated, and the cell monolayer was washed with prewarmed sterile phosphate buffered saline (PBS). Cells were transfected with the appropriate plasmids using LipofectAMINE™ 2000 Reagent (Invitrogen) according to the manufacturer's protocol. The cells were harvested at different time points. Western blot analysis or other experiments were performed.

For the establishment of stable transfectants of pTER-β-catenin, after transfection, HeLaT cells were selected with 200 μg/ml Zeocin, and resistant clones were mixed and cultured. Zeocin-resistant clones were tested for their ability to downregulate β-catenin by Western blot and RT-PCR. The resulting resistant clones populations were designated HeLaT-β-catenin-RNAi.

### Isolation of total RNA

Total RNA was extracted from cells by using TRIZOL Reagent (Invitrogen) according to the standard protocol. The integrity of the RNA samples was determined by electrophoresis through agarose gels and staining with ethidium bromide, and the 18S and 28S RNA bands were visualized under UV light. RNA was stored at -80°C in RNase-free water until reverse transcription or fluorescence labeling.

### Western blot analyze and RT-PCR

HeLaT-β-catenin-RNAi cells were grown in the presence or absence of DOX (2 μg/ml). For Western blot analysis, cells were harvested at different time points and lysed in lysis buffer, then Western blot analysis was performed with the use of conventional protocols as described previously [14]. In brief, total proteins were separated by SDS-PAGE, then transferred to nitrocellulose membranes (PROTRAN). The antibodies and dilutions used included anti-β-catenin (C19220; 1:5000; BD Transduction Laboratories), anti-β-actin (AC-15; 1:5000; Sigma), and after extensive washing the membranes were incubated with anti-mouse IgG-horseradish peroxidase conjugate antibody (Zhongshan Company) for 1 h at room temperature and developed with a Luminol chemiluminescence detection kit (Santa Cruz). Membranes were reprobed for β-actin to normalize for loading and to allow for accurate quantification. Protein expression was quantified using a Gel EDAS 293 analysis system (Cold Spring USA Corporation) and Gel-Pro Analyzer 3.1 software (Media Cybernetics).

For RT-PCR, total RNA (5 μg) was used for cDNA synthesis by reverse transcription using mouse-mammary tumor virus (M-MLV) Reverse Transcriptase (Promega), according to the manufacturer's protocol in a total volume of 25 μl. All PCR reactions were performed using standard PCR conditions: 95°C 5 min, 95°C 1 min, annealing at different temperatures for each gene respectively 1 min (Table 1), extension 72°C 1 min for 30 cycles, and a final extension at 72°C for 10 min. The PCR products were visualized by electrophoresis in 2% agarose gels, followed by staining with ethidium bromide, and quantified using a Gel EDAS 290 analysis system (Cold Spring USA Corporation) and Gel-Pro Analyzer 3.1 software (Media Cybernetics). Primer sequences are listed in Table 1.

### Reporter assay

To measure the transcriptional activity of β-catenin/TCF, a luciferase reporter assay was performed using the TCF reporter constructs TOPFLASH and FOPFLASH. Cells were replated and transfected in 24-well plates, with either TOPFLASH or FOPFLASH (100 ng) and the internal control plasmid pRL-TK (5 ng) using LipofectAMINE™ 2000 Reagent (Invitrogen). The TOPFLASH and FOPFLASH reporters contain two sets of three copies of wild-type or mutant β-catenin/TCF binding sites respectively as well as the Thymidine Kinase (TK) minimal promoter upstream of the Firefly Luciferase open reading frame. After transfection, cells were treated with or without DOX (2 μg/ml) for 3 days. Then, luciferase activity was determined using the Dual-luciferase reporter assay system (Promega). Firefly luciferase activity was normalized to Renilla luciferase activity. All results are expressed as means ± SD for independent triplicate cultures.

### TdT-mediated dUTP nick end labeling (TUNEL) assay

Apoptotic cells were confirmed with the *in situ *cell death detection kit, Alkaline Phosphatase (Roche Applied Science), in accordance with the manufacturer's instructions. In brief, HeLaT-β-catenin-RNAi cells were grown on coverslips. The next day, cells were treated with or without DOX (2 μg/ml) for 3 days. Coverslips with adherent cells were fixed in 4% paraformaldehyde for 1 h at room temperature and permeabilized with 0.1% Triton X-100 for 2 min on ice. DNA fragments were labeled with the TUNEL reaction mixture for 60 min at 37°C in a humidified atmosphere in the dark. The coverslips were then incubated with Converter alkaline phosphatase for 30 min at 37°C in a humidified chamber, rinsed in PBS, and incubated with nitro blue tetrazolium/5-bromo-4-chloroindol-3-yl phosphate (Roche Applied Science) for 10 min. Cells were mounted cell side downward on a microscope slide, and the apoptotic cells (dark blue staining) were counted under a microscope. Three fields were randomly counted for each sample.

### Microarray designing and expression profiling

A total of 1384 apoptosis-related genes were selected. Probes against these genes were designed with OligoArray2_1 (University of Michigan). The oligonucleotide probes were synthesized and spotted as described previously [15]. The oligonucleotide microarray covers 1384 apoptosis-related sequences and some controls. For the microarray analysis, 50–100 μg of DNA-free total RNA from control or induced cells was reverse transcribed and labeled with Cy3 or Cy5 and then hybridized to the oligonucleotide microarray. Data acquisition and data analysis were performed using a GenePix 4000B scanner and GenePix Pro 5.1 software (Axon Instruments). Detailed information about the oligonucleotide microarray production, microarray analysis and data is available at http://gpcrome.cbi.pku.edu.cn:2005/chip.

### Statistical analysis

Expression ratios of the analysed genes were calculated comparing genes' expression values of HeLaT-β-catenin-RNAi cells treated with or without DOX(see Additional file1) A 1.5-fold or higher level of target genes' expression ratio in at least three of the five repeated experiments was considered. SPSS for Windows 10.0 (SPSS Inc.) was used to analyze the data. Two-tailed unpaired Student's *t *test was used to compare the statistical significance of the differences in data from two groups. Values of *P *< 0.05 were considered statistically significant (see Additional file 2). Moreover, referenced data from SAM was calculated (see Additional file 3).

## Results

### Inducible reduction of β-catenin expression in stable pTER transfectants

**Figure 1:**
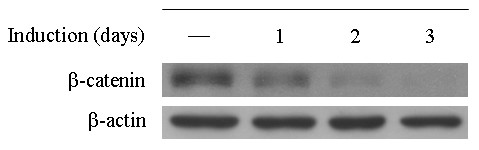
**Reduction of β-catenin expression in stable pTER transfectants**. Exponentially proliferating HeLaT-β-catenin-RNAi cells were treated with DOX and whole cell lysates were prepared at the time points indicated. Total cell lysates were separated by SDS-PAGE and immunoblotted with an antibody to β-catenin, expression levels were normalized for loading by probing for β-actin.

**Figure 2:**
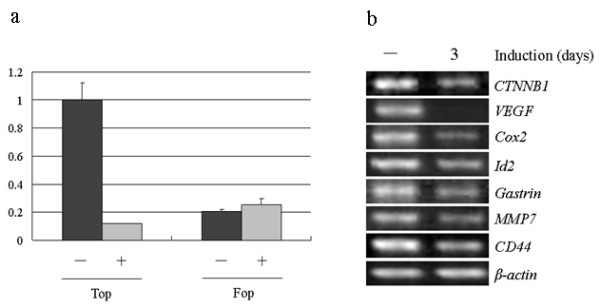
**Downregulation of β-catenin/TCF-driven transcription on knockdown of β-catenin**. **(a) **HeLaT-β-catenin-RNAi cells were transfected with TOPFLASH or FOPFLASH luciferase reporter construct. After 3 days with or without DOX treatment, luciferase reporter gene expression was determined. The pRL-TK *Renilla *luciferase reporter construct was co-transfected in each sample to normalize transfection efficiency. The activity of the reporter luciferase is expressed relative to the activity in control cells, which is defined as 1.0. All experiments were performed in triplicate and are expressed as means and SD. **(b) **Relative expression levels of known β-catenin/TCF target genes in the HeLaT-β-catenin-RNAi cells following treatment with DOX, as measured by semi-quantitative RT-PCR. *β-actin *expression is used as a control.

T-REx™-HeLa (HeLaT) cells that stably expressed the tetracycline (Tet) repressor were transfected with pTER-β-catenin and selected with Zeocin. Stable transfectants were analyzed for β-catenin expression by Western blot and RT-PCR before and after DOX induction. The protein level of β-catenin was reduced within 1 day after treatment and completely suppressed on the third day (Figure 1). Meanwhile, mRNA level of β-catenin was also significantly reduced after 3 days of treatment as demonstrated by RT-PCR (Figure 2b) and microarray analysis (Figure 4). The inhibitory effect was shown to be specific because treatment of the parental HeLaT cells with DOX did not alter β-catenin levels (data not shown). In addition, expression of β-actin was not affected by DOX, showing the specificity of the knockdown. These data indicate that the stably integrated inducible RNAi vector could effectively suppress β-catenin expression and result in prolonged decreases in specific cellular gene expression without marked effects on other cellular proteins.

### Downregulation of β-catenin/TCF-driven transcription on knockdown of β-catenin

We then investigated the effects of β-catenin knockdown on the transcriptional activity of β-catenin/TCF [14]. The luciferase reporters TOPFLASH and FOPFLASH, which have a minimal Thymidine Kinase (TK) promoter and either wild type (TOP) or mutated (FOP) binding sites for the β-catenin/TCF complex, have been widely used to characterize the transcriptional activity of β-catenin/TCF [16]. These reporter constructs were transfected into HeLaT-β-catenin-RNAi cells, and luciferase activity was determined after 3 days of treatment with DOX.

We found that the spontaneous activity of the TCF reporter, TOPFLASH, was reduced to background (FOPFLASH) levels on reduction of β-catenin levels by the induced expression of pTER-β-catenin by DOX (Figure 2a). There was little effect of induction on the FOPFLASH reporter (Figure 2a).

To determine whether downregulation of β-catenin in this manner leads to decreased expression of β-catenin/TCF regulated genes, we investigated the effect of induction on the expression of several cellular genes known to be regulated by β-catenin/TCF. The endogenous mRNA expression levels of *Vascular endothelial growth factor *(*VEGF*) [17], cyclooxygenase-2 (*Cox2*) [18], *Inhibitor of differentiation protein 2 *(*Id2*) [19], *Gastrin *[20], *Matrix metalloproteinase-7 *(*MMP7*) [21,22] and *CD44 *[23], but not *β-actin*, were all reduced after treatment with DOX (Figure 2b; Table 1).

These results indicate that inducible knockdown of β-catenin by the stably integrated RNAi vector pTER-β-catenin in HeLaT cells results in the downregulation the β-catenin/TCF-dependent gene expression.

### Induction of apoptosis by RNAi depletion of β-catenin

**Figure 3:**
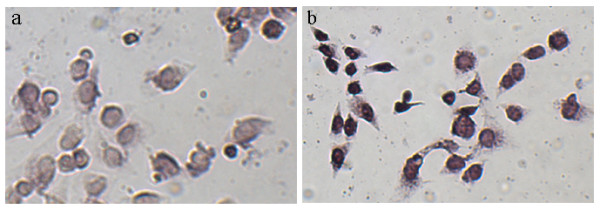
**Apoptosis induced by depletion of β-catenin**. HeLaT-β-catenin-RNAi cells were grown on coverslips and treated without (**a**) or with (**b**) DOX. After 3 days, cells were analyzed for apoptosis with the TUNEL assay. Dark-blue staining of nuclei indicates apoptosis.

Previous studies demonstrated that inhibiting the activity of Wnt/β-catenin pathway induced apoptosis [6]. To determine whether depletion of β-catenin by inducible RNAi could promote the death of cells, TUNEL assay was performed. HeLaT-β-catenin-RNAi cells were treated with or without DOX (2 μg/ml) for 3 days. These cells were then analyzed by TUNEL assay (Figure 3). About 38% cells were TUNEL-positive in the DOX group (Figure 3b), compared with 13% in the control group (Figure 3a) (*P *< 0.01). These data suggested that depletion of β-catenin by RNAi in HeLaT-β-catenin-RNAi cells made the cells more sensitive to apoptosis.

### Analysis and validation of oligonucleotide microarray assay following decreased expression of β-catenin

The data we show above and previous studies suggest that Wnt/β-catenin pathway may be associated with cellular apoptosis. However, the molecular mechanisms by which Wnt/β-catenin pathway regulate apoptosis are unclear until now. Chen *et al*. found that Wnt-mediated β-catenin/TCF transcription was responsible for providing protection against apoptosis [7]. To systematically investigate Wnt/β-catenin regulated genes at the genome-wide scale that contribute to the apoptosis effect, oligonucleotide microarray analysis was performed. The HeLaT-β-catenin-RNAi cells were induced with DOX. After 3 days, 50–100 μg of DNA-free total RNA from control or induced cells was reverse transcribed and labeled with Cy3 or Cy5 and then hybridized to the oligonucleotide microarray containing 1384 apoptosis-related genes (Additional Table 1). Microarray experiments were performed comparing induced *vs*. control HeLaT-β-catenin-RNAi cells. Duplicate experiments were carried out on a single total RNA preparation from the cells.

**Figure 4:**
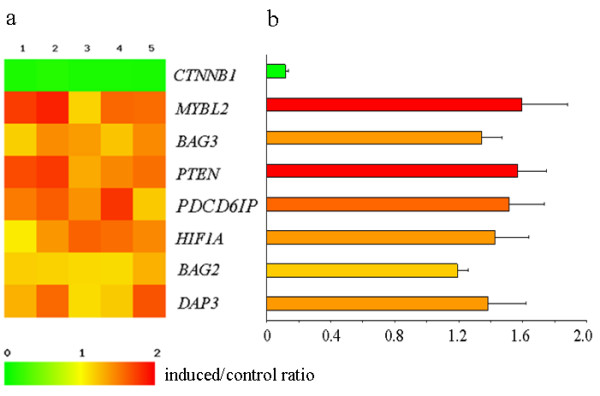
**Microarray cluster analysis demonstrating differentially expressed genes in HeLaT-β-catenin-RNAi cells treated with DOX**. After 3 days of treatment, 50–100 μg of DNA-free total RNA from control or induced cells was reverse transcribed and labeled with Cy3 or Cy5 and then hybridized to the oligonucleotide microarray containing 1384 apoptosis-related genes. Microarray experiments were performed comparing induced *vs*. control cells. Shown here are the intensity ratios of 5 separate experiments **(a) **or means ± S.D **(b)**. The color bar represents the intensity ratio of induced *vs*. control cells.

In this study, 130 differential expression genes due to reduced expression of β-catenin were identified (Table 2; Additional Table 2). Figure 4 and Table 2 show the alteration levels of several differential expression genes in control *vs*. induced cells. We found that the mRNA level of β-catenin (*CTNNB1*) was markedly reduced after induction with DOX, demonstrating the effectiveness of the RNAi system. Meanwhile, several apoptosis-related genes, such as *MYBL2*, *BAG3*, *PTEN*, *PDCD6IP*, *HIF1A*, *BAG2 *and *DAP3*, were significantly upregulated (*P *< 0.05) (Figure 4; Table 1 and 2).

**Figure 5:**
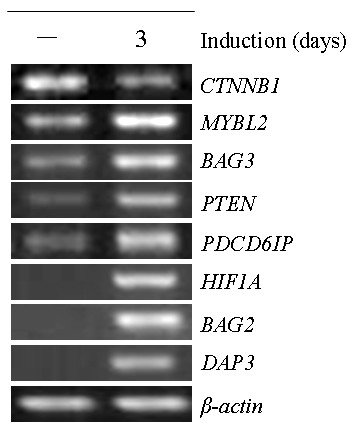
**Validation of oligonucleotide microarray results of 8 selected genes by semi-quantitative RT-PCR**. HeLaT-β-catenin-RNAi cells were treated with or without DOX for 3 days. RT-PCR was performed as described in **Methods**. Amplified fragments of *CTNNB1*, *MYBL2*, *BAG3*, *PTEN*, *PDCD6IP*, *HIF1A*, *BAG2 *and *DAP3 *genes are indicated. *β-actin *gene was used as an internal control and was amplified from the same samples.

To determine the gene expression level of specific Wnt/β-catenin regulated genes, semi-quantitative RT-PCR analysis was used. A panel of 8 genes, randomly selected among the 130 identified by microarray analysis, was analyzed. We confirmed this by RT-PCR downregulation of *CTNNB1 *and upregulation of *MYBL2*, *BAG3*, *PTEN*, *PDCD6IP*, *HIF1A*, *BAG2 *and *DAP3 *(Figure 5; Table 1). All of these genes showed a comparable alteration between microarray assay and semi-quantitative RT-PCR analysis. Our data suggest that some of these apoptosis-related genes may be regulated by Wnt/β-catenin pathway and involved in the molecular mechanisms by which Wnt/β-catenin pathway regulates apoptosis.

## Discussion

Wnt/β-catenin pathway is involved in various differentiation events during embryonic development and leads to a range of diseases, most notably cancer, when aberrantly activated [3-5]. It has been demonstrated that this pathway not only plays a role in the promotion of cell proliferation and cell cycle progression, but also may provide an important survival function to facilitate cell transformation. Inhibition of the activity of Wnt/β-catenin pathway induces apoptosis in some cancer cell lines [24-36]. Meanwhile, activation of the Wnt/β-catenin pathway inhibits apoptosis in some lines [37-41]. In addition, de la Taille *et al*. demonstrated that Wnt/β-catenin pathway plays a role in the progression of human prostate cancer, especially to the acquisition of apoptosis-resistant phenotype [42]. In spite of these findings, the molecular mechanisms by which Wnt/β-catenin pathway exerts its effect on cellular apoptosis are not understood.

In our study, the HeLaT cells that stably expressed the Tet repressor were transfected with pTER-β-catenin and selected using Zeocin [11]. The resulting stable transfectants were analyzed for β-catenin expression by Western blot and RT-PCR before and after DOX induction. The mRNA and protein levels of β-catenin were significantly reduced after 3 days of treatment. We then investigated the effects of β-catenin knockdown on TCF reporter activity. The spontaneous activity of the TCF reporter (TOPFLASH) was reduced to background levels on reduction of β-catenin levels by the induced expression of pTER-β-catenin by DOX. Meanwhile, the expression levels of several cellular genes known to be regulated by β-catenin/TCF were all reduced after treatment with DOX. These results indicate that after induction by DOX, not only the expression levels of β-catenin, but the transcriptional activity of β-catenin/TCF is significantly inhibited in HeLaT cells. We also found that depletion of β-catenin in this manner made the cells more sensitive to apoptosis by TUNEL assay. However, Cobas *et al*. demonstrated that there was no evidence of induced apoptosis in bone marrow progenitors when β-catenin gene was inactivated by an inducible Cre-loxP-mediated system [43]. This suggests that the effects observed in our current study are likely to be cell type specific.

It is well known that Wnt/β-catenin pathway regulates the transcription of a suite of genes controlling numerous aspects of development and human diseases, ranging from cellular proliferation, differentiation and apoptosis [3,4]. However, little is known about how Wnt/β-catenin pathway regulates the expression of apoptosis-related genes. Microarray technology provides a tool to detailed study the regulation of gene expression [44]. To further elucidate the role of Wnt/β-catenin pathway in apoptosis, we designed a microarray covering 1384 apoptosis-related genes. The 130 differentially regulated genes presented in this expression profiling analysis were identified using stringent selection criteria and are candidates for direct or indirect targets of Wnt/β-catenin pathway, which may play critical roles in tumorigenesis in certain tumors. This set of regulated genes is highly significant due to statistical procedures (*t *test, *P *< 0.05). The significance of the oligonucleotide microarray expression data is further supported by RT-PCR. Confirmation of 8 regulated genes by RT-PCR provides experimental support for the reliability of the microarray data.

The overall pattern of gene expression observed in response to the inhibition of Wnt/β-catenin pathway by inducible RNAi vector against β-catenin has important implications for elucidating the role of this pathway in apoptosis. Of particular interest are those genes that have been implicated in several cell-survival pathways, such as Phosphatase and tensin homolog (PTEN)-Phosphatidylinositol 3-kinase (PI3K)-AKT pathway [45-47], NF-κB pathway [48,49] and p53 pathway [50]. For example, *PTEN*, which negatively regulates the PI3K-AKT survival pathway, was upregulated after DOX treatment. Elevated expression of *PTEN *may inhibit the activity of PI3K-AKT pathway and induce these cells to apoptosis. Consistent with our results, it has been demonstrated that Wnt pathway regulates cellular apoptosis by PI3K-AKT pathway [37,40]. Similarly, *NFKBIA*, the gene coding for IκBα, which retains NF-κB dimers in the cytoplasm to prevent the activation of NF-κB pathway [49], was also upregulated after induction. Also, inhibition of NF-κB pathway induces apoptosis [48,49]. This is consistent with the report from Bournat *et al*. They demonstrated that expression of Wnt-1 increases survival of PC12 cells in the absence of serum by activating the anti-apoptotic factor NF-κB [51]. We also found that a p53-binding gene *TP53BP1 *and a p53-induced gene *TP53I11 *were elevated after the inhibited activity of Wnt/β-catenin by treatment. The 53BP1 encoded by *TP53BP1 *gene was found to be able to bind p53 protein and enhanced p53-mediated transcriptional activation [52,53]. Meanwhile, *TP53I11*, a direct p53 target gene, was proved to induce cell apoptosis and enhance the apoptotic effects of arsenic trioxide [54]. These data suggest that inhibition the Wnt/β-catenin pathway by pTER-β-catenin may lead to elevated p53 activity, which can induce apoptosis under some circumstances [50]. Other pro-apoptotic genes, such as *Programmed cell death 5 *(*PDCD5*) [55], *Death-associated protein 3 *(*DAP3*) [56] and *Fas-associated via death domain *(*FADD*) [57], were all significantly up-regulated after induction of RNAi against β-catenin by DOX and may play a role in promoting apoptosis of these cells. Paradoxically, papers from Jablons's group suggest that an inhibitor of apoptosis family protein, Survivin, may play a role in mediating the functions of Wnt/β-catenin pathway in apoptosis [27,30,31]. However, in our system, the alteration of Survivin expression before and after induction is not significant (data not shown). Thus, these results indicate that Wnt/β-catenin mediated transcription may regulate other anti-apoptosis molecules yet to be identified. In our system, most of the differentially expressed genes are upregulated after inhibition of Wnt/β-catenin pathway. Since inhibition of Wnt/β-catenin pathway should lead to the downregulation of its direct target genes, we believe that most of the differentially expressed genes are most likely the indirect target genes of Wnt/β-catenin pathway. The precise mechanism needs further investigation. The data obtained from microarray suggest that inhibition of Wnt/β-catenin pathway induces apoptosis at least in part through upregulating the expression of several pro-apoptotic genes, which may be involved in some important cell-apoptotic pathways, such as PTEN-PI3K-AKT pathway, NF-κB pathway and p53 pathway.

As evident from the examples above, assessment of differentially expressed genes with known functions is useful to monitor pathways or biologic processes that are triggered in expression profiling experiments. In addition, microarray expression profiling of β-catenin-decreasing cells identified at least 13 regulated genes that are, so far, not annotated with an experimentally verified function, predicted biologic process or molecular function. This provides a basis for further experimental studies to provide more direct information on gene function.

## Conclusion

In summary, we successfully downregulate the expression of β-catenin and also the transcriptional activity of Wnt/β-catenin pathway in HeLa cells in a stably inducible manner. Microarray data suggest that a series of pro-apoptotic genes, which may be involved in some important cell-apoptotic pathways, such as PTEN-PI3K-AKT pathway, NF-κB pathway and p53 pathway may contribute to the enhanced apoptosis. Further studies are needed to identify the precise mechanism underlying the Wnt/β-catenin pathway in apoptosis.

## Abbreviations

APC = adenomatous polyposis coli; DAP3 = death-associated protein 3; DOX = doxycycline; FADD = Fas-associated via death domain; GSK3β = glycogen synthesis kinase 3β; PDCD5 = programmed cell death 5; PI3K = phosphatidylinositol 3-kinase; PTEN = phosphatase and tensin homolog; RNAi = RNA interference; siRNA = small interfering RNA; TCF = T cell factor; TUNEL = TdT-mediated dUTP nick end labeling.

## Competing interests

The author(s) declare that they have no competing interests.

## Authors' contributions

**WYH **established the stable transfectants of pTER-β-catenin, performed western blot and reporter assays, and wrote the manuscript. **HML **designed, annotated the microarray, performed the statistical analysis and constructed website. **SDC**, **YYB **and **WSQ **were responsible for construction of microarray. **ZHX **performed the RNA extraction. **ZW **and **YSB **performed the RT-PCR assay. **QLP **and **BJF **conducted the cell culturing. **CQ **provided some ideas and suggestions on dealing the parental T-REx™-HeLa cells. **XNZ **and **LSG **designed the study and were corresponding authors. All authors read and approved the final manuscript.

## Pre-publication history

The pre-publication history for this paper can be accessed here:



## Supplementary Material

Additional File 1 **all microarray data and annotation**. The data provided all probes annotation in the microarray and expression values Click here for file

Additional File 2 **130 differential expressed genes from microarray**. The data provided differential expressed genes with ID, name, location and p-values. Click here for file

Additional File 3 **SAM result of microarray data**. The data was producted by SAM Click here for file

## Figures and Tables

**Table 1:** RT-PCR primer sequences and annealing temperatures for validation of microarray expression data.

**Gene symbol**	**PCR primer sequences**	**Annealing (°C)**	**PCR-based expression**	**Chip-based expression**
*CTNNB1*	1 st CCTGGTGCTGACTATCCAGT	52	Down	Down
	2nd ACTTCCACACATGATCACAT			
*VEGF*	1 st GAGGGCAGAATCATCACGAA	62	Down	NA
	2nd AACGCTCCAGGACTTATACC			
*Cox2*	1 st CGAGGTGTATGTATGAGTGT	60	Down	NA
	2nd AGGGAGTCGGGCAATC			
*Id2*	1 st CCGCTCGAGACCATGAAAGCCTTCAGTCCCGTGAGG	61	Down	NA
	2nd CGGGATCCTTGCCACACAGTGCTTTGCTG			
*Gastrin*	1 st CGGAATTCACCATGCAGCGACTATGTG	58	Down	NA
	2nd CGGGATCCATGTTCTCATCCTCAGCACT			
*MMP7*	1 st TGTTAAACTCCCGCGTCATA	58	Down	NA
	2nd GCGTTCATCCTCATCGAAGT			
*CD44*	1 st CCATCCTCGTCCCGTCCT	56	Down	NA
	2nd GCCCTTCTATGAACCCATACCT			
*MYBL2*	1 st CTCATCTTGTCCTGAGGTGT	56	Up	Up
	2nd TGAGGCCAGGCCTGACGTG			
*BAG3*	1 st AGCCATCCTGGAGAAGGTAC	56	Up	Up
	2nd GGCACCCATCTCCATGATTG			
*PTEN*	1 st CCACCACAGCTAGAACTTA	52	Up	Up
	2nd CAACAGTGCCACTGGTCTA			
*PDCD6IP*	1 st CTCGGCTCTGTTAGTGTAAC	52	Up	Up
	2nd CACTTCTGACTAGTTCCTCT			
*HIF1A*	1 st GATACCAACAGTAACCAACCT	52	Up	Up
	2nd CCGGTTTAAGGACACATTCT			
*BAG2*	1 st GAAGATCTATGGCTCAGGCGAAGAT	56	Up	Up
	2nd ATTTGCGGCCGCCTAATTGAATCTGCTTTCAGC			
*DAP3*	1 st CCTCCTAGTGGCCGTGGATG	56	Up	Up
	2nd GCGTTACTTAGGAACAGCAG			
*β-actin*	1 st GGCGGCACCACCATGTACCCT	56	Control	NA
	2nd AGGGGCCGGACTCGTCATACT			

Genes marked "NA" were not included in the 130 differentially regulated genes (listed in Additional Table 2); "Down" and "Up" refer to the expression levels in induced *vs*. control cells.

**Table 2:** List of representative genes upregulated in the HeLaT-β-catenin-RNAi cells following treatment with DOX.

**Gene symbol**	**Accession**	**Gene description**	**Ratio mean**^a^	***P *value**
*AATF*	GenBank:NM_012138	apoptosis antagonizing transcription factor	1.583515	0.000483
*BAG2*	GenBank:NM_004282	BCL2-associated athanogene 2	1.1912	0.01584154
*BAG3*	GenBank:NM_004281	BCL2-associated athanogene 3	1.340984	0.03940599
*CTNNB1*	GenBank:NM_001904	catenin (cadherin-associated protein), beta 1,88kDa	0.116545	0.00000433
*DAP3*	GenBank:NM_033657	death associated protein 3	1.382345	0.02268848
*FADD*	GenBank:NM_003824	Fas (TNFRSF6)-associated via death domain	1.272306	0.01998745
*HIF1A*	GenBank:NM_001530	hypoxia-inducible factor 1, alpha subunit(basic helix-loop-helix transcription factor)	1.425380	0.01105092
*MYBL2*	GenBank:NM_002466	v-myb myeloblastosis viral oncogene homolog (avian)-like 2	1.594979	0. 3012066
*NFKBIA*	GenBank:NM_020529	nuclear factor of kappa light polypeptide gene enhancer in B-cells inhibitor, alpha	1.266071	0.01633079
*PDCD5*	GenBank:NM_004708	programmed cell death 5	1.429819	0.00544860
*PDCD6IP*	GenBank:NM_013374	programmed cell death 6 interacting protein	1.515707	0.02611401
*PTEN*	GenBank:NM_000314	phosphatase and tensin homolog (mutated in multiple advanced cancers 1)	1.568053	0.00204383
*TP53BP1*	GenBank:NM_005657	tumor protein p53 binding protein 1	1.272044	0.00446432
*TP53I11*	GenBank:NM_006034	tumor protein p53 inducible protein 11	1.343337	0.00532453

^a^Shown here are the means of the intensity ratios of 5 separate experiments by comparing induced *vs*. control cells.
